# Comparative Efficacy of Danshen Class Injections for Treating Acute Coronary Syndrome: A Multidimensional Bayesian Network Meta-Analysis of Randomized Controlled Trials

**DOI:** 10.3389/fphar.2020.01260

**Published:** 2020-08-26

**Authors:** Siyu Guo, Jiarui Wu, Mengwei Ni, Shanshan Jia, Jingyuan Zhang, Wei Zhou, Xinkui Liu, Miaomiao Wang, Xiaomeng Zhang

**Affiliations:** Department of Clinical Chinese Pharmacy, School of Chinese Materia Medica, Beijing University of Chinese Medicine, Beijing, China

**Keywords:** Danshen class injections, acute coronary syndrome, network meta-analysis, randomized controlled trials, outcomes research

## Abstract

**Background:**

Acute coronary syndrome, that is a common and serious cardiovascular disease, imposes a huge economic burden on global public health. And Danshen class injections are commonly used in the treatment of acute coronary syndrome in China. Thus, the Bayesian network meta-analysis was devised to investigate the efficacy of different Danshen class injections against acute coronary syndrome.

**Methods:**

Eligible inclusion and exclusion criteria were established in advance. Then, a systematic literature search was performed in several databases from inception to February 2020. Further, the included randomized controlled trials data were adopted to calculation, prepare graphs and multidimensional cluster analysis by WinBUGS 1.4.3, Stata V.13.0 and R 3.6.1 software, respectively.

**Results:**

A total of 53 eligible randomized controlled trial studies with 6401 patients were obtained that evaluated the clinical effectiveness rate, the level of hypersensitive C-reactive protein, C-reactive protein, interleukin-6, fibrinogen, and adverse reactions after the application of Danshen class injections plus western medicine. Compared with western medicine alone, Danshen class injections combined with western medicine therapy were associated with significantly improved the therapeutic effect. In addition, the results of the multidimensional cluster analysis demonstrated that Danhong injection + western medicine and Danshen injection + western medicine had better therapeutic effects. However, since most eligible randomized controlled trial studies did not focus on the monitoring of adverse reactions, the safety of these Chinese herbal injections needs to be further explored.

**Conclusion:**

Based on this Bayesian network meta-analysis results, Danhong injection + western medicine and Danshen injection + western medicine might have a better impact on acute coronary syndrome patients. Nevertheless, more large samples, high-quality clinical and multicenter randomized controlled trial studies should be tested and verified in the future.

## Introduction

Acute coronary syndrome (ACS) is a medical emergency (Kosuge and Kimura, 2016). The underlying mechanism of ACS is triggered by atheromatous plaque enlargement, instability, and rupture or erosion (Amsterdam et al., 2014; Saremi, 2017). ACS includes ST elevation myocardial infarction (STEMI), unstable angina (UA) and non-ST elevation myocardial infarction (NSTEMI) (Hedayati et al., 2018). In 2014, it is estimated that in the United States, 633,000 persons were diagnosed with acute coronary syndrome (Virani et al., 2020). Additionally, the risk of ACS is greater with age (Timmis, 2015). Presently, the treatments for ACS mainly rely on anti-platelet, anti-coagulant, anti-ischemic, thrombolytic and percutaneous coronary intervention (PCI) (Wu et al., 2015). However, although clopidogrel is an effective antiplatelet drug in patients with ACS, it has the limitations of high bleeding risk and stent thrombosis (Wang et al., 2018). Previous studies reported that PCI may be related to vascular endothelial cell dysfunction, platelet aggregation and neutrophil obstruction, which will lead to restenosis of the stent or some major adverse cardiac events (MACE) (Farooq et al., 2011; Kirtane et al., 2014).

To find more effective and promising therapeutic approaches to manage ACS, traditional Chinese medicine (TCM) combined with western medicine (WM) have received widespread attention from clinicians in China (Zhang et al., 2020). In the theory of TCM, the pathogenesis of ACS is closely correlated to stagnant blood block (Wu et al., 2015). Furthermore, Chinese herbal injections (CHIs) are a new type of TCM formulation with high bioavailability and fast action (Li et al., 2017). According to accumulated evidenced-based data, the combination of CHIs and WM is associated with considerable efficacy in treating patients with ACS (Zhao et al., 2018). For example, a systematic review reported that Danhong injection (DH) could improve the therapeutic efficacy and reduce the incidence of MACE events in ACS patients after PCI (Zou et al., 2018). Moreover, systematic reviews and meta-analysis studies shown that Sodium Tanshinone IIA Sulfonate injection (STS) and Danshenchuanxiongqin injection (DSCXQ) combined with WM appeared to be efficacious in the treatment UA (Zhang et al., 2016; Tan et al., 2018).

In China and Japan, Danshen (*Radix Salviae Miltiorrhizae*) is the dried root or rhizome of *Salvia miltiorrhiza Bunge*, commonly used to treat cardiovascular-related diseases (Meim et al., 2019; Yuan et al., 2020). The relative studies validated that Danshen possesses the characteristics of relaxing coronary artery muscle, improving microcirculation, reducing blood viscosity and inhibiting thrombogenesis (Cheng, 2006). The CHIs containing the extract of Danshen are termed Danshen class injections (DSCIs) (Liu et al., 2019). In this context, this study included 8 DSCIs, namely, Danshen injection (DS), Fufang Danshen injection (FFDS), Danhong injection (DH), Dansenduofensuanyan injection (DSDFSY), Danshen Salvianolic Acids injection (DSSA), Danshenchuanxiongqin injection (DSCXQ), Sodium Tanshinone IIA Sulfonate injection (STS) and Guanxinning injection (GXN). Compared with the traditional double-arm meta-analysis, network meta-analysis (NMA) can synthesize different interventions for the same disease and perform direct or indirect comparisons (Grant, 2019; Sun et al., 2019; Zhang et al., 2019). Moreover, NMA can assist assessing and ranking the therapeutic effects of different treatments (Rouse et al., 2017; Pan et al., 2019). However, at present, there are many systematic review studies on the adjuvant treatment of ACS with a certain type of DSCIs, lacking of comparison of efficacy between different types of DSCI (Wu et al., 2015; Zhang et al., 2016; Wu et al., 2017; Tan et al., 2018; Zou et al., 2018). Therefore, the present research adopted NMA to explore the comparative effectiveness and safety between different DSCIs plus WM against ACS, providing a reference for clinical practice.

## Materials and Methods

The procedure of the current NMA research was conducted based on the Preferred Reporting Items for Systematic Reviews and Meta-Analyses (PRISMA) guidelines (Hutton et al., 2015) (**Supplementary File 1**).

### Eligibility and Exclusion Criteria

The Eligibility criteria for this study conformed the PICOS framework, namely, participants, interventions, comparisons, outcomes and study design. So, randomized controlled trials (RCTs) that met the following criteria were included in this study: (1) Study design. Only RCTs mentioned in articles were enrolled. (2) Participants: The current NMA included patients were diagnosed with ACS, and without limitations on race, age, gender, or nationality. (3) Interventions and comparisons: The experiment group was treated by CHIs (DS, FFDS, DH, DSDFSY, DSSA, DSCXQ, STS, and GXN) combined with WM. Patients in the control group were both received WM treatment or WM plus another CHIs, excluding PCI. The commonly used WM drugs were primarily nitrate, low molecular weight heparin, aspirin, nifedipine, statins, β-receptor blocker, and so on. There are no restrictions on dosage and duration of treatment. The corresponding treatment measures should be taken for patients with other complications. (4) Outcomes. The primary outcomes of this study were the clinical effectiveness rate. According to clinical symptoms and objective indicators, the effectiveness status was divided into effective, effective, and ineffective. The clinical effectiveness rate = (number of total patients − number of invalid patients)/number of total patients∗100%. It was regarded as invalidation when the clinical symptoms and electrocardiogram didn’t change or worsen, or the frequency of chest pain wasn’t decreased or increased (Ministry of Health of People’s Republic of China, 1993). Additionally, the secondary outcomes were hypersensitive C-reactive protein (hs-CRP), C-reactive protein (CRP), interleukin-6 (IL-6), fibrinogen (FIB) and adverse reactions (ADRs). Previous research evidences suggested that local and systemic inflammation play a pivotal role in the pathogenesis of ACS (Liuzzo, 2001; Elhajj et al., 2004). For example, the increase of IL-6, hs-CPR and CPR concentration can be observed in patients with systemic signs of systemic inflammation (Stefanadis et al., 2000; Centurión, 2016; Nanchen et al., 2019). In addition, FIB is the main determinant of thrombus formation, and its increase is related to the degree of coronary atherosclerosis, especially in patients with ACS (de Moerloose et al., 2010; Kurtul et al., 2016; Buljubasic et al., 2017).

RCTs were excluded if any of the following conditions were accord: (1) The therapy in RCTs includes PCI, or other TCM preparations. (2) The article data is wrong or incomplete. (3) The article could not be obtained full text. (4) Data duplicates. (5) No related outcome.

### Search Strategy

Studies concerning RCTs published from inception to February 2020 were identified through a systematic electronic search of the following seven databases: the China National Knowledge Infrastructure Database (CNKI), the Chinese Scientific Journals Full-text Database (VIP), the Wan-Fang Database, the Chinese Biomedical Literature Database (SinoMed), the Cochrane Library, PubMed and Embase. For this purpose, the free-text keywords and MESH (Medical Subject Heading) terms were 1utilized, including “acute coronary syndrome [MeSH Terms]”, “Acute Coronary Syndromes”, “Coronary Syndrome, Acute”, “Coronary Syndromes, Acute”, “Syndrome, Acute Coronary”, “Syndromes, Acute Coronary”, “Danshen”, “Compound danshen”, “Xiangdan”, “Fufangdanshen”, “Danhong”, “Beitong”, “Salvianolate”, “Salvianolic acid”, “Danshenduofensuan”, “Danshenchuanxiongqin”, “Sodium tanshinone IIA sulfonate”, “Guanxinning” and “randomized controlled trial [Publication Type]”. In addition, there was no restrictions on blinding methods, publication year and language. Furthermore, the references of related articles were manually reviewed to identify further studies. The detailed and specific retrieval strategies are shown in **Supplementary File 2**.

### Data Extraction and Quality Assessment

Data of eligible studies were independently extracted by two reviewers (SYG and MWN) by using Microsoft Excel (Microsoft Corp, Redmond, WA). The specifically designed form captured information on the study characteristics, including publication data (publication date, title and authors’ names), details of patients’ characteristics (sample sizes, age and sex), intervention (the types of CHIs, dose and duration), outcomes (the primary and secondary outcomes) and factors to evaluate risk of bias.

According to the Cochrane risk of bias tool (Higgins et al., 2011), two investigators (SJ and JZ) independently conducted the quality assessment of all included RCTs. Each trial was scored as low, high, or unclear risk of bias on 7 quality evaluation items, including random sequence generation (selection bias), allocation concealment (selection bias), blinding of participants and personnel (performance bias), blinding of outcome assessment (detection bias), incomplete outcome data (attrition bias), selective reporting (reporting bias) and other bias. When discordance occurred in the process of literature selection, data extraction and quality assessment, the final results were resolved by adjudication with a third reviewer (WZ) or consensus.

### Statistical Analysis

In consideration of the clinical and within-study and between-study heterogeneity among the included RCTs, the random-effect model was selected. Moreover, according to the Bayesian hierarchical model and Markov Chain Monte Carlo algorithm, WinBUGS 1.4.3 (MRC Biostatistics Unit, Cambridge, UK) software was adopted to analyze data. For dichotomous outcomes, the combined results were presented as odds ratios (ORs) with 95% confidence intervals (95% CIs). For continuous outcomes, the results were calculated as the mean differences (MDs) with 95% CIs. If 95% CIs of ORs did not include 1 and 95% CIs of MDs did not contain 0, the differences between the groups would be considered statistically significant (Huang et al., 2019). In the WinBUGS program, we set 200,000 iterations, and the first 10 000 iterations were regarded as burn-in for annealing to eliminate the influence of the initial value (Crainiceanu and Goldsmith, 2010).

Furthermore, the Stata V.13.0 software was utilized (Stata Corporation) to generate graphs in order to compare the relationship between different treatment measures. The surface under the cumulative ranking (SUCRA) area was employed to rank the probabilities for different interventions under each outcome. The SUCRA ranges from 0 to 100%, assigning to the worst and best therapeutic measures, respectively (Cope and Jansen, 2013; Trinquart et al., 2016). In addition, the publication bias of included RCTs were checked though a comparison adjusted funnel plot (Chaimani et al., 2013).

### Multidimensional Cluster Analysis

The multidimensional cluster analysis was performed to assess comprehensive efficacy of competing treatments in any three outcomes based on SUCRA value. Hence, the “scatterplot3d” package in R 3.6.1 software (Mathsoft, Cambridge,USA) was used for multidimensional clustering analysis in this study. These interventions were clustered by using the k-means method, and the number of clusters was modified according to the actual situation (Ball and Hall, 1967; Haldar et al., 2008; Ahlqvist et al., 2018). The steps of clustering were as follows: (1) The included interventions were randomly divided into k initial categories, and the average of the outcome indicators of these k categories were regarded as the initial aggregation points. (2) An intervention was classified as the closest aggregation point category, and then the aggregation point of that category was updated to the mean of the present outcome indicators. All interventions were re-categorized and classified and repeat step (2) until they have been assigned. Finally, the ranking of treatment interventions was visually displayed through a three-dimensional stereogram. The different colors dots in the figure represent different types of interventions.

## Results

### Literature Selection

**Figure 1:**
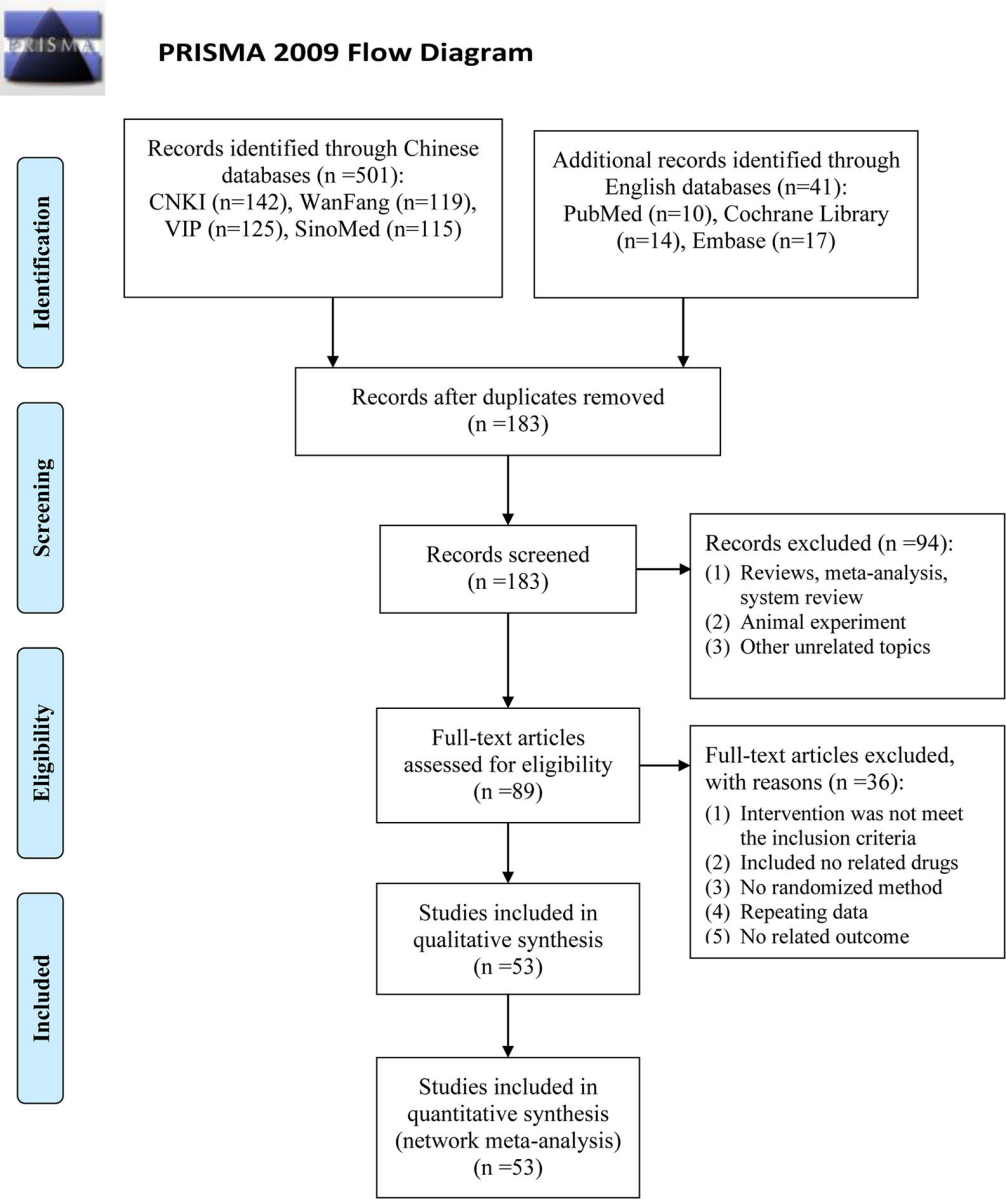
Flow diagram of eligible literature selection. (CNKI, the China National Lnowledge Infrastructure Database; VIP, the Chinese Scientific Journals Full-text Database; SinoMed, the Chinese Biomedical Literature Database; n, number of publications).

**Figure 2:**
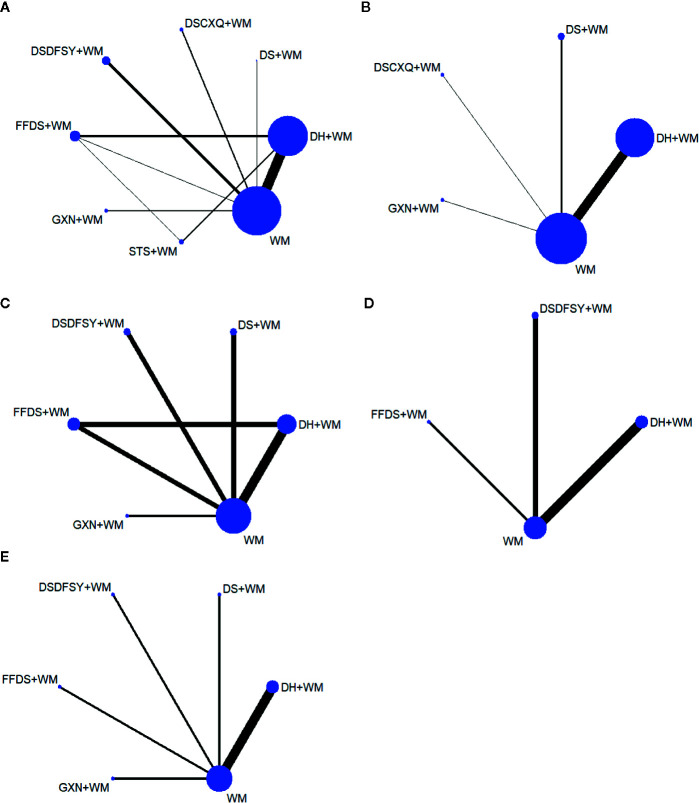
Network graphs for different outcomes. **(A)** clinical effectiveness rate; **(B)** the level of hs-CRP; **(C)** the level of CRP; **(D)** the level of IL-6; **(E)** the level of FIB. (DS, Danshen injection; FFDS, Fufang Danshen injection; DH, Danhong injection; DSDFSY, Dansenduofensuanyan injection; DSCXQ, Danshenchuanxiongqin injection; STS, Sodium Tanshinone IIA Sulfonate injection; GXN, Guanxinning injection; WM, western medicine; hs-CRP, hypersensitive C-reactive protein; CRP, C-reactive protein; IL-6, interleukin-6; FIB, fibrinogen).

A total of 542 articles were obtained by searching 7 databases. After removing the duplicates, 183 articles need to be screened for the title and abstract. Then, 94 studies were excluded because of reviews, meta-analysis, system reviews, animal experiment and other irrelevant literature. Ultimately, 53 RCTs studies conducted in China from 2007 to 2019 were eligible in this NMA. The details of the articles screening process are depicted in **Figure 1**. Seven types of DSCIs were incorporated, including DS, FFDS, DH, DSDFSY, DSCXQ, STS and GXN. The network graphs of the above 7 CHIs with different outcomes are described in **Figure 2**. The detailed information on CHIs shown in **Supplementary File 3**.

### Study Characteristics

Overall, the 53 eligible RCTs studies involved 6,401 patients (3,175 in the control group, and 3,226 in the experiment group), and most of them were middle-aged and elderly. With the exception of five studies that did not report gender composition, male patients accounted for approximately 52.73% of the total study population. A total of nine comparisons were evaluated: DS + WM vs. WM (n = 5), DSCXQ + WM vs. WM (n = 2), DSDFSY + WM vs. WM (n = 5), DH + WM vs. WM (n = 30), FFDS + WM vs. WM (n = 2), GXN + WM vs. WM (n = 4), DH + WM vs. FFDs + WM (n = 3), DH + WM vs. STS + WM (n = 1), and DH + WM/STS + WM vs. FFDS + WM (n = 1). The therapies of the control group were WM, primarily including nitrate, low molecular weight heparin, aspirin, nifedipine, statins, β-receptor blocker, etc. Moreover, the duration of treatment reported by most studies was 14 days. The details of the included study characteristics are shown in **Table 1**.

**Table 1:** Characteristics of the studies included in this AMA.

Study ID	Sample size		Sex (M/F)		Average age		Therapy of experiment group	Menstruum	Therapy of control group	Course (days)	Outcomes
	E	C	E	C	E	C					
Li et al., 2007	37	36	39/34		67.4 ± 6.5		DS20ml+WM	NR	WM	14d	②
Shi, 2013	30	30	34/26		63.5± 7.4		DS20ml+WM	0.9%NS 480mL	WM	NR	①⑤
Long, 2011	35	35	35/35		67		DS+WM	NR	WM	14d	②
Ma and Liang, 2016	60	60	30/30	32/28	72.22 ± 7.18	71.88 ± 7.04	DS20ml+WM	5%GS 250mL	WM	14d	③
Su and Zhao, 2017	59	59	33/26	31/28	69.46 ± 6.67	69.44 ± 6.65	DS20ml+WM	NR	WM	14d	③
Wu et al., 2009	50	48	28/22	27/21	54∼79	52∼80	DSCXQ10ml+WM	5%GS/NS 500mL	WM	10d	①②⑥
Zhang et al., 2014	713	800	371/342	433/367	43~77	39~74	DSCXQ10ml+WM	NR	WM	14d	①⑥
Lin and Yu, 2017	45	44	27/18	24/20	67.1 ± 6.7	66.9 ± 6.1	DSDFSY200mg+WM	5%GS 250mL	WM	14d	①⑥
Xia et al., 2017	31	35	20/11	22/13	61.5 ± 11.4	62.1 ± 12.6	DSDFSY200mg+WM	0.9%NS 250 mL	WM	14d	①④
Li et al., 2019	54	54	29/25	30/24	60.17 ± 5.14	60.01 ± 5.28	DSDFSY200mg+WM	5%GS 250mL	WM	1m	①③④
Li, 2013	24	22	14/10	12/10	66 ± 8	60 ± 5	DSDFSY200mg+WM	5%GS 250mL	WM	14d	①③⑤⑥
Lin, 2015	30	30	39/21		67.2 0 ± 7.18		DSDFSY200mg+WM	10%GS 250mL	WM	14d	①
Wang and Zhang, 2012	43	43	28/15	29/14	76~91	76~88	DH20ml+WM	5%GS/0.9%NS 250mL	WM	14d	①③⑤
Xu, 2011	42	40	25/17	22/18	42-78	41-76	DH40ml+WM	5%GS 250mL	WM	14d	①
Li, 2015	90	90	60/30	58/32	67.8 ± 3.2	68.5 ± 4.1	DH20ml+WM	5%GS/0.9%NS 250mL	WM	14d	①③⑤
Liu et al., 2008	150	150	101/49	98/52	57.1 ± 6.2	55.6 ± 7.1	DH40ml+WM	5%GS/0.9%NS 250mL	WM	14d	①⑥
Ding, 2012	30	30	13/17	12/18	66.50 ± 7.10	65.90 ± 7.30	DH40ml+WM	0.9%NS 250 mL	WM	14d	①
Yang, 2010	40	40	43/37		58.85 ± 6.8		DH20ml+WM	NS 250 mL	WM	14d	①
Du and Chen, 2009	45	44	54/35		68.4 ± 7.2		DH20ml+WM	5%GS 250mL	WM	14d	③⑥
Zhou, 2011	30	30	NR		NR		DH20mg+WM	5%GS/NS 250mL	WM	14d	①②⑥
Nie and Zhao, 2012	30	30	41/19		42-88		DH40ml+WM	5%GS/NS 250mL	WM	15d	②
Su and Jiang, 2012	43	43	54/32		63.2 ± 10.7		DH40ml+WM	NS 250 mL	WM	14d	②
Gu et al., 2011	70	70	NR		NR		DH40ml+WM	5%GS 250mL	WM	14d	③⑤
Liang et al., 2019	35	35	21/14	22/13	62.8± 5.3	63.1± 5.5	DH40ml+WM	0.9%NS 250 mL	WM	14d*2	②④
Cao et al., 2010	47	35	30/17	21/14	71.5 ± 4.5	69.9 ± 7.2	DH40ml+WM	5%GS	WM	14d	⑤
Feng, 2011	30	30	NR		NR		DH20mg+WM	5%GS/NS 250mL	WM	14d	①②⑥
Wei, 2012	61	61	73/49		59.4 ± 5.2		DH20ml+WM	NR	WM	10d	①②
Guan et al., 2010	60	60	NR		73.54 ± 10.12	72.15 ± 10.03	DH30ml+WM	NS 250 mL	WM	14d	②④
Zhang et al., 2010	65	60	34/31	32/28	65.4 ± 4.6	65.8 ± 4.3	DH30ml+WM	5%GS/NS 250mL	WM	28d	②④⑥
Chen, 2012	30	30	31/29		50~65		DH40ml+WM	5%GS/NS 250mL	WM	14d	①⑥
Chen and Wang, 2011	45	45	52/38		67 ± 10.2		DH40ml+WM	5%GS/NS 250mL	WM	14d	①⑥
Li et al., 2010	42	38	43/37		58.3 ± 8.7		DH30ml+WM	5%GS/NS 250mL	WM	14d	②
Li et al., 2007	20	20	29/11		M : 66 ± 9.5 F : 65 ± 6.6		DH20mL+WM	NR	WM	14d	①
Li and Song, 2011	34	32	22/10	18/11	60.12 ± 4.51	58.46 ± 6.25	DH20~30mL+WM	NS 250 mL	WM	14d	②
Du, 2011	30	30	NR		NR		DH20mg+WM	5%GS/NS 250mL	WM	14d	①⑥
Li and Meng, 2008	26	22	16/10	13/9	56~82	49 ± 76	DH40ml+WM	5%GS/NS 500mL	WM	14d	①⑥
Wang et al., 2015	123	122	82/41	80/42	83.4 ± 6.4	83.2 ± 7.2	DH40ml+WM	5%GS/NS 250mL	WM	14d	①⑥
Lao et al., 2013	35	35	19/16	18/17	62.27 ± 2.29	63.58 ± 2.25	DH20mL+WM	5%GS/NS 250mL	WM	56d	②⑥
Fei et al., 2010	60	60	NR		73.54 ± 10.12	72.15 ± 10.03	DH30mL+WM	NS 250 mL	WM	14d	④
Zhang et al., 2012	44	44	22/22	26/18	22~41	20~44	DH20mL+WM	10%NS 250 mL	WM	10d	①⑥
He, 2014	36	36	22/14	20/16	56. 8 ± 9.4	57. 1 ± 9.8	DH+WM	5%GS/NS 250mL	WM	14d	①
Li and Ge, 2011	44	42	50/36		64.5 ± 8		DH20mL+WM	10%GS/NS 250mL	WM	10d	①②⑥
Hong et al., 2004	90	30	52/38	17/13	62.95 ± 9.36	66.00 ± 8.96	FFDS20ml+WM	5%GS/NS 250mL	WM	14d	③④⑤
Ruan et al., 2017	33	33	15/18	14/19	54.38 ± 6.73	52.93 ± 6.80	FFDS20ml+WM	5%GS 250mL	WM	1m	①③⑥
Xia and Wang, 2011	54	54	35/19	33/21	64 ± 13		GXN20ml+WM	5%GS/0.9%NS 250mL	WM	30d	①⑥
Liao et al., 2013	45	45	NR		NR		GXN20ml+WM	5%GS/NS 250mL	WM	14d	③⑤
Zhao, 2011	60	60	36/24	38/22	45~80	48~81	GXN20ml+WM	5%GS 250mL	WM	14d	①⑥
Li et al., 2016	38	35	25/13	21/14	61.85 ± 9.76	60.97 ± 9.3	GXN20ml+WM	5%GS 250mL	WM	28d	②
Xiao, 2009	39	40	65/14		83.35 ± 7.46		DH20mL+WM	5%GS/0.9%NS 250mL	FFDS20ml+WM	30d	①③⑥
Cui, 2008	33	33	28/5	33/26	78.3 ± 2.4	77.9 ± 2.8	DH20mL+WM	5%GS 250mL	FFDS20ml+WM	14d	①⑥
Cai et al., 2011	69	67	42/27	44/23	56 ± 7	57 ± 10	DH20mL+WM	NR	FFDS20ml+WM	10d	①③⑥
Yang and Zhang, 2010	48	48	30/18	29/19	45~82		STS50mg+WM	5%GS/0.9%NS 250mL	DH30ml+WM	15d	①⑥
Hou, 2013	A:34 B:35	30	A: 26/8 B:25/10	23/7	A: 43~69 B: 41~68	44~67	A: DH40mL+WM B: TST+WM	5%GS/0.9%NS 250mL	FFDS20ml+WM	14d	①

E, experimental group; C, control group; M, male; F, female; NR, not reported; WM, western medicine, such as low molecular weight heparin, aspirin, nifedipine, statins and β-receptor blocker; GS, glucose solution; NS, NaCl solution; m, month; d, day; ①, clinical effectiveness rate; ②, hs-CRP; ③, CRP; ④, IL-6; ⑤, FIB; ⑥, ADRs.

### Quality Evaluation

**Figure 3:**
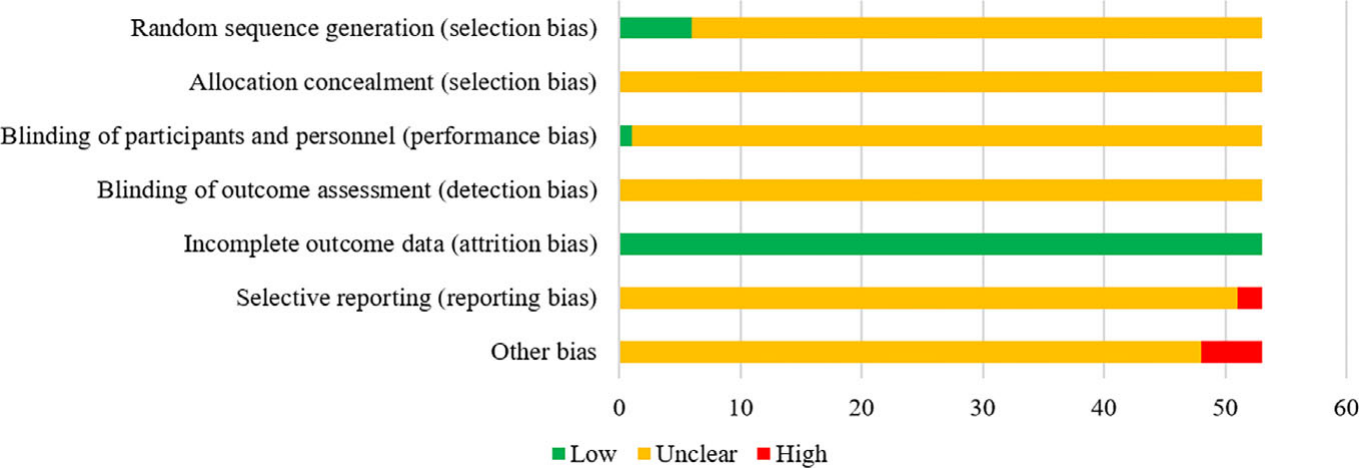
The risk of bias of the included RCTs. The vertical axis represents the quality evaluation items and horizontal axis represents the number of RCTs.

In terms of random sequence generation (selection bias), a total of 6 RCTs used a random number table to generate random sequences, so they were considered to be low risk. Regarding performance bias, 1 RCT study mentioned the use of the single blind method, so it was evaluated as low risk. In terms of attrition bias, all studies had no incomplete data, so the evaluation was “low risk”. Regarding reporting bias, 2 studies reported incomplete outcome indicators mentioned in the design plan, so they were regarded as high risk. In terms of other bias, five studies did not report whether the experiment group and the control group were comparable at baseline, which might affect the study results, so they were rated as high risk. In addition, the risk bias entries of the remaining studies were considered “unclear” due to insufficient information. In general, the quality of included studies was not high. The risk of bias for each eligible RCTs is depicted in **Figure 3**.

### Outcomes

#### Clinical Effectiveness Rate

A total of 34 RCTs referred to the clinical effectiveness rate (DS + WM vs. WM (n = 1), DSCXQ + WM vs. WM (n = 2), DSDFSY + WM vs. WM (n = 5), DH + WM vs. WM (n = 18), FFDS + WM vs. WM (n = 1), GXN + WM vs. WM (n = 2), DH + WM vs. FFDs + WM (n = 3), DH + WM vs. STS + WM (n = 1), and DH + WM/STS + WM vs. FFDS + WM (n = 1)), including 7 types of CHIs. Apart from DS and FFDS injection, other CHIs combined with WM were superior to WM alone, and the difference was statistically significant. **Table 2** shows the following specific outcomes: DH + WM vs. WM (OR= 0.21, 95% CIs: 0.15–0.29), DSDFSY + WM vs. WM (OR= 0.24, 95% CIs: 0.12–0.45), DSCXQ + WM vs. WM (OR= 0.57, 95% CIs: 0.27–0.90), STS + WM vs. WM (OR= 0.31, 95% CIs: 0.12–0.82) and GXN + WM vs. WM (OR= 0.32, 95% CIs: 0.13–0.76). In addition, compared with the efficacy of FFDS + WM, DH + WM (OR= 2.96, 95% CIs: 1.71–5.20) and DSDFSY + WM (OR= 2.63, 95% CIs: 1.07–6.49) were related to significantly improved clinical outcomes. There was no statistically significant difference between other interventions.

**Table 2:** Odds ratio/mean difference (95%CIs) of all therapeutic measures.

Comparison	Clinical effectiveness rate	hs-CRP	CRP	IL-6	FIB
DS+WM vs					
FFDS+WM	0.34 (0.03, 1.99)	——	−0.34 (−6.01, 4.66)	——	−0.56 (−6.23, 5.26)
DH+WM	1.02 (0.11, 5.50)	−1.56 (−11.00, 9.00)	0.33 (−4.21, 5.02)	——	−0.56 (−5.02, 3.98)
DSDFSY+WM	0.90 (0.09, 5.23)	——	−1.10 (−5.84, 3.53)	——	−0.76 (−6.04, 4.65)
DSCXQ+WM	0.39 (0.04, 2.27)	1.37 (−10.92, 13.58)	——	——	——
STS+WM	0.68 (0.06, 4.65)	——	——	——	——
GXN+WM	0.67 (0.07, 4.48)	−2.29 (−13.59, 9.53)	−0.52 (−5.72, 4.59)	——	−0.37 (−5.72, 5.07)
WM	0.21 (0.02, 1.12)	−3.08 (−12.48, 7.28)	−2.18 (−5.85, 1.39)	——	−0.99 (−5.03, 3.18)
FFDS+WM vs					
DH+WM	**2.96 (1.71, 5.20)**	——	0.60 (−1.98, 4.57)	−2.63 (−51.87, 46.86)	0.01 (−4.48, 4.37)
DSDFSY+WM	**2.63 (1.07, 6.49)**	——	−0.76 (−5.39, 4.50)	−2.77 (−48.31, 42.31)	−0.18 (−5.53, 5.11)
DSCXQ+WM	1.12 (0.52, 2.87)	——	——	——	——
STS+WM	2.02 (0.77, 5.27)	——	——	——	——
GXN+WM	1.97 (0.68, 6.03)	——	−0.20 (−5.23, 5.65)	——	0.17 (−5.18, 5.53)
WM	0.62 (0.33, 1.17)	——	−1.87 (−5.42, 2.42)	−4.93 (−49.18, 38.96)	−0.44 (−4.49, 3.58)
DH+WM vs					
DSDFSY+WM	0.88 (0.43, 1.86)	——	−1.41 (−5.75, 2.49)	−0.10 (−23.09, 22.02)	−0.21 (−4.06, 3.79)
DSCXQ+WM	0.38 (0.21, 0.81)	2.85 (−4.43, 9.85)	——	——	——
STS+WM	0.68 (0.27, 1.70)	——	——	——	——
GXN+WM	0.67 (0.25, 1.78)	−0.68 (−6.89, 5.30)	−0.83 (−5.74, 3.66)	——	0.18 (−3.74, 4.22)
WM	**0.21 (0.15, 0.29)**	−**1.43 (**−**3.24,** −**0.25)**	−2.51 (−5.47, 0.13)	——	−0.45 (−2.24, 1.42)
DSDFSY+WM vs					
DSCXQ+WM	0.43 (0.19, 1.09)	——	——	——	——
STS+WM	0.77 (0.24, 2.41)	——	——	——	——
GXN+WM	0.76 (0.25, 2.30)	——	0.56 (−4.18, 5.45)	——	0.39 (−4.57, 5.35)
WM	**0.24 (0.12, 0.45)**	——	−1.11 (−4.05, 1.94)	−2.12 (−11.43, 7.12)	−0.22 (−3.73, 3.19)
DSCXQ+WM vs					
STS+WM	1.79 (0.54, 5.15)	——	——	——	——
GXN+WM	1.75 (0.57, 4.89)	−3.59 (−12.64, 5.75)	——	——	——
WM	**0.57 (0.27, 0.90)**	−4.36 (−11.26, 2.76)	——	——	——
STS+WM vs					
GXN+WM	0.98 (0.27, 3.72)	——	——	——	——
WM	**0.31 (0.12, 0.82)**	——	——	——	——
					
GXN+WM vs					
WM	**0.32 (0.13, 0.76)**	−0.80 (−6.70, 5.13)	−1.68 (−5.48, 2.14)	——	−0.62 (−4.18, 2.91)

The bolded and underlined results indicate statistical significance.

**Figure 4:**
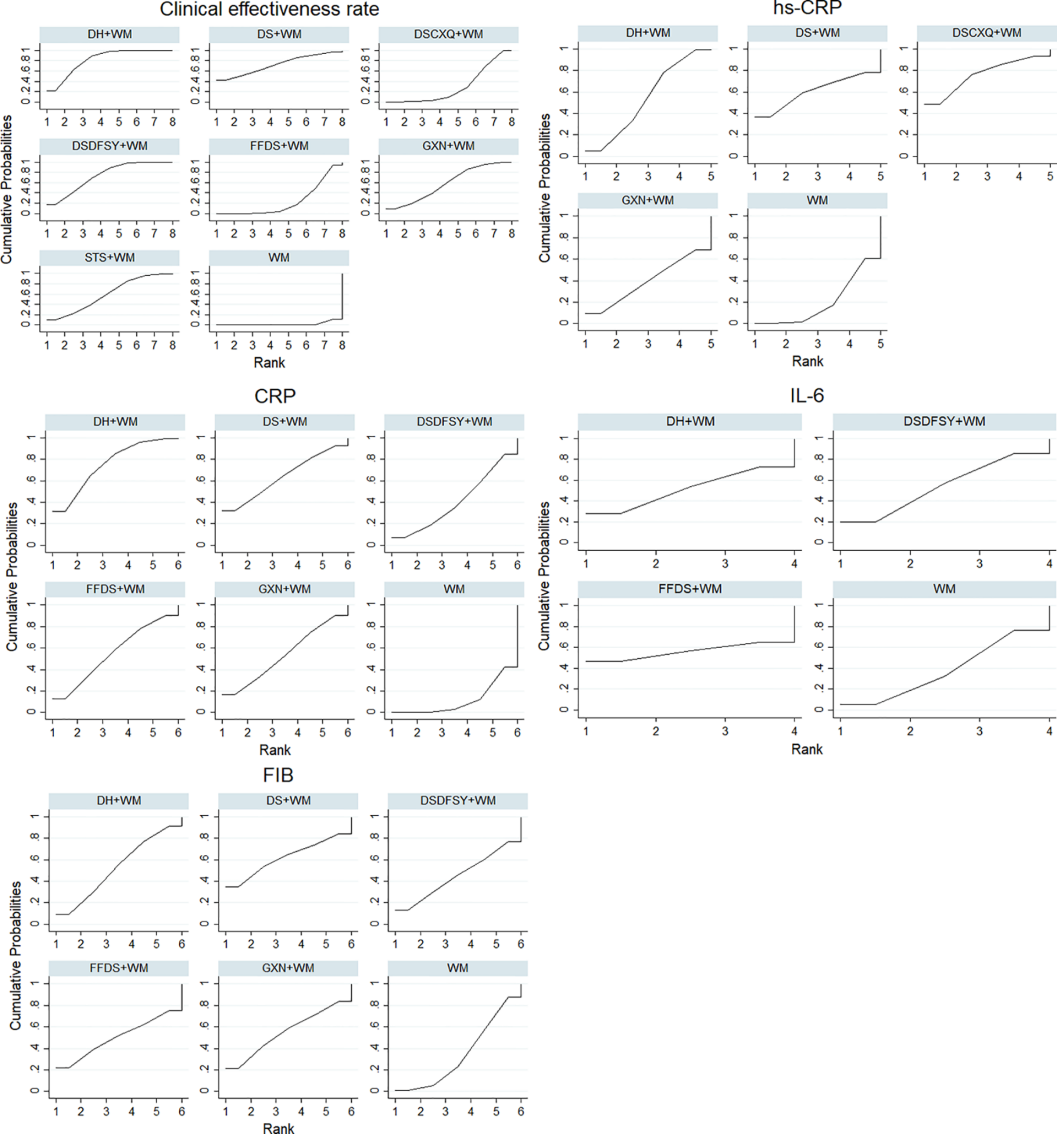
SUCRA for different outcomes. The vertical axis represents cumulative probabilities and horizontal axis represents rank. (DS, Danshen injection; FFDS, Fufang Danshen injection; DH, Danhong injection; DSDFSY, Dansenduofensuanyan injection; DSCXQ, Danshenchuanxiongqin injection; STS, Sodium Tanshinone IIA Sulfonate injection; GXN, Guanxinning injection; WM, western medicine; hs-CRP, hypersensitive C-reactive protein; CRP, C-reactive protein; IL-6, interleukin-6; FIB, fibrinogen).

According to the ranking of SUCRA probabilities (**Figure 4**, **Table 3**), DH + WM (81.8%) was the most likely to become the best intervention for improving the clinical effectiveness rate. Moreover, the other 6 CHIs were ranked as follows: DSDFSY+WM (73.4%) > DS+WM (72.1%) > STS+WM (59.3%) > GXN+WM (58.3%) > DSCXQ+WM (29.9%) > FFDS+WM (23.4%).

**Table 3:** SUCRA (%) of all therapeutic measures.

Intervention	Clinical effectiveness rate	hs-CRP	CRP	IL-6	FIB
DS+WM	72.1	60.5	63.7	——	62
FFDS+WM	23.4	——	55	56.2	50.2
DH+WM	81.8	54.1	75.3	51.6	52.5
DSDFSY+WM	73.4	——	40.7	54	45.2
DSCXQ+WM	29.9	76.1	——	——	——
STS+WM	59.3	——	——	——	——
GXN+WM	58.3	39.4	53.7	——	55.4
WM	1.8	19.9	11.7	38.2	34.7

The redder color in the table means the higher the ranking of the corresponding interventions.

#### The Level of hs-CRP

A total of 16 RCTs involving 4 interventions investigated hs-CRP [DS + WM vs. WM (n = 2), DSCXQ + WM vs. WM (n = 1), DH + WM vs. WM (n = 12) and GXN + WM vs. WM (n = 1)]. The specific outcomes are described in **Table 2**. DH+WM (MD= −1.43, 95% CIs: −3.24 ~ −0.25) could achieve a better effect in hs-CRP than WM alone, and the difference was statistically significant. Simultaneously, there was no statistically significant difference between other interventions. Base on the data of SUCRA probabilities (**Figure 4**, **Table 3**), the 4 CHIs were ranked as follows: DSCXQ+WM (76.1%) > DS+WM (60.5%) > DH+WM (54.1%) > GXN+WM (39.4%).

#### The Level of CRP

In total, 13 RCTs with 5 CHIs observed data regarding CRP [DS + WM vs. WM (n = 2), DSCXQ + WM vs. WM (n = 2), DH + WM vs. WM (n = 4), FFDS+WM (n = 2), GXN+WM (n = 1) and DH + WM vs. FFDs + WM (n = 2)]. The results demonstrated that no significant differences, which are shown in **Table 2**. According to the SUCRA values (**Figure 4**, **Table 3**), the 5 CHIs were ranked as follows: DH+WM (75.3%) > DS+WM (63.7%) > FFDS+WM (55%) > GXN+WM (53.7%) > DSDFSY+WM (40.7%).

#### The Level of IL-6

A total of 7 RCTs with 3 CHIs presented data about IL-6 [DSDFSY + WM vs. WM (n = 2), DH + WM vs. WM (n = 4) and FFDS+WM (n = 1)]. Additionally, the results demonstrated that no significant differences in IL-6 was observed among the above CHIs, which are shown in **Table 2**. In terms of the SUCRA values (**Figure 4**, **Table 3**), the 3 CHIs were ranked as follows: FFDS+WM (56.2%) > DSDFSY+WM (54%) > DH+WM (51.6%).

#### The Level of FIB

The data on the FIB were available for 8 RCTs including 5 types of CHIs [DS + WM vs. WM (n=1), DSDFSY + WM vs. WM (n = 1), DH + WM vs. WM (n = 4), GXN+WM vs. WM (n=1) and FFDS+WM (n = 1)]. Simultaneously, the results showed that no significant differences among above CHIs groups for FIB (**Table 2**). According to the SUCRA values (**Figure 4**, **Table 3**), the 5 CHIs were ranked as follows: DS+WM (62%) > GXN+WM (55.4%) > DH+WM (52.5%) > FFDS+WM (50.2%) > DSDFSY+WM (45.2%).

#### Safety

In terms of safety, 28 studies did not address ADRs, and 17 studies indicated that there were no significant ADRs. Meanwhile, 1 study (Li Y M, 2007) only reported monitoring ADRs related to liver and kidney dysfunction and bleeding tendency, but did not report the corresponding results. In addition, 1 study (Yang and Zhang, 2010) only described 3 cases of ADRs in the experiment group (STS+WM) and 2 cases in the control group (DH+WM), but none of them affected the treatment. In Yang’s research (Lin and Yu, 2017), 3 cases (allergic rash, vomiting and bloating) and 2 cases (vomiting) of ADRs occurred in the experiment group (DSDFSY+WM) and the control group (WM), respectively, and the symptoms were mild. In Lao’s study (Lao et al., 2013), one patient in the control group (WM) had elevated transaminase and returned to normal levels after hepatoprotective treatment; none of the experiment group (DH+WM) had abnormal liver and kidney test indicators during the treatment process, and there were no serious ADRs. In Ruan’s study (Ruan et al., 2017), 4 ADRs occurred in the experiment group (FFDS+WM), including drowsiness, fever and mild gastrointestinal reactions; 5 ADRs occurred in the control group (WM), containing dizziness, drowsiness and mild gastrointestinal reactions. In the research of Xia and Whang (2011), 1 patient in the experiment group (GXN+WM) presented with facial redness and mild headache, and 2 had rashes.; the control group (WM) developed 2 cases of flushing and mild headache, 1 case of rash, and 1 case of local swelling by injection. In the study Zhao (2011), some patients in the experiment group (GXN+WM) and the control group (WM)developed headaches, which were relieved by reducing nitrate dosage. In the research of Xiao (2009), 1 patient in the experimental group (DH+WM) developed facial redness and mild headache, while 2 patients in the control group (FFDS+WM) developed a sense of local swelling from the injection. However, since most eligible RCTs studies did not focus on the monitoring of ADRs, the safety of these CHIs needs to be further explored.

### Multidimensional Cluster Analysis

**Figure 5:**
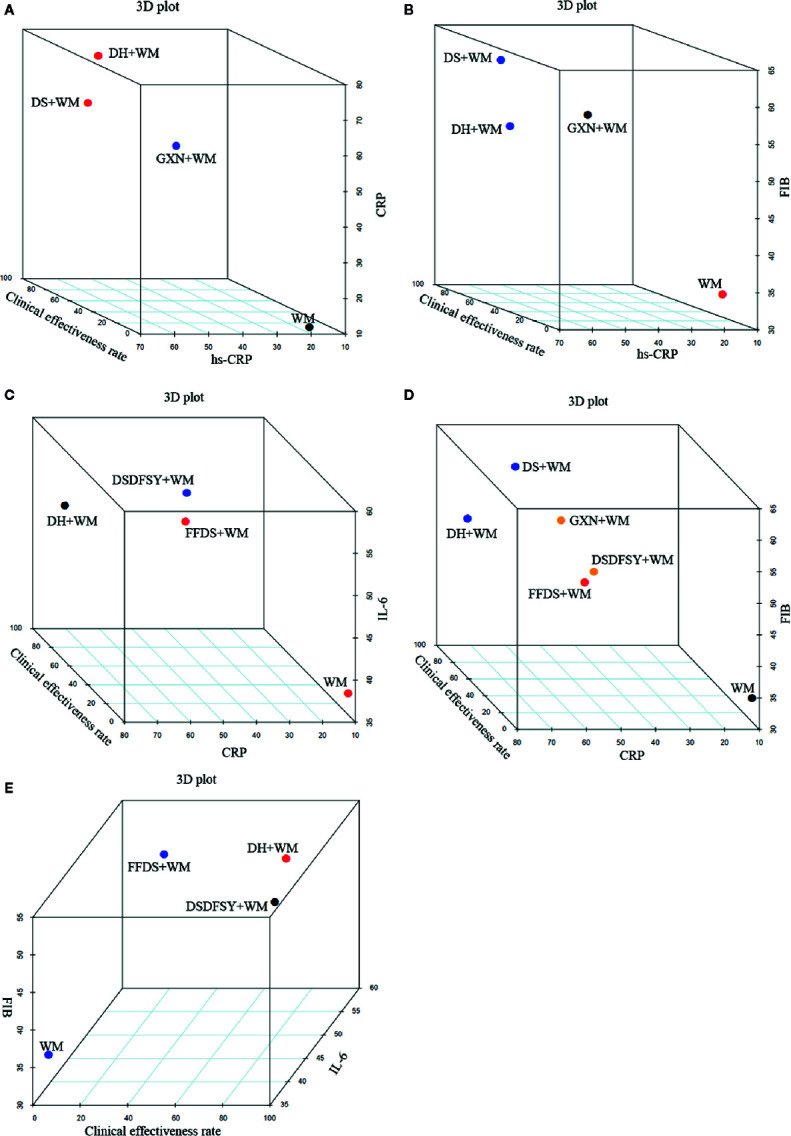
Multidimensional cluster analysis plots. The different colors dots represent different types of interventions. **(A)** hs-CRP (X axis), clinical effectiveness rate (Y axis) and CRP (Z axis); **(B)** hs-CRP (X axis), clinical effectiveness rate (Y axis) and FIB (Z axis); **(C)** CRP (X axis), clinical effectiveness rate (Y axis) and IL-6 (Z axis); **(D)** CRP (X axis), clinical effectiveness rate (Y axis) and FIB (Z axis); **(E)** IL-6 (X axis), clinical effectiveness rate (Y axis) and FIB (Z axis). (DS, Danshen injection; FFDS, Fufang Danshen injection; DH, Danhong injection; DSDFSY, Dansenduofensuanyan injection; DSCXQ, Danshenchuanxiongqin injection; STS, Sodium Tanshinone IIA Sulfonate injection; GXN, Guanxinning injection; WM, western medicine; hs-CRP, hypersensitive C-reactive protein; CRP, C-reactive protein; IL-6, interleukin-6; FIB, fibrinogen).

When cluster analysis was conducted to 4 interventions that reported the hs-CRP, clinical effectiveness rate and CRP, DH+WM was superior to the other regimens, and WM only was evaluated the worst (**Figure 5A**). In terms of the clinical effectiveness rate, hs-CRP and FIB, DS + WM and DH + WM were similarly superior (**Figure 5B**). Furthermore, Regarding the clinical effectiveness rate, CRP and IL-6, DH+WM was similarly preferred (**Figure 5C**). Moreover, DS + WM and DH + WM were dominant in the comprehensive ranking of the clinical effectiveness rate, CRP and FIB (**Figure 5D**). With regard to the comprehensive ranking of the clinical effectiveness rate, FIB and IL-6, the results indicated that DH + WM had the potential to be the best intervention (**Figure 5E**). In summary, the results of the multidimensional cluster analysis demonstrated that DH+WM and DS+WM might have better therapeutic effects.

### Publication Bias

**Figure 6:**
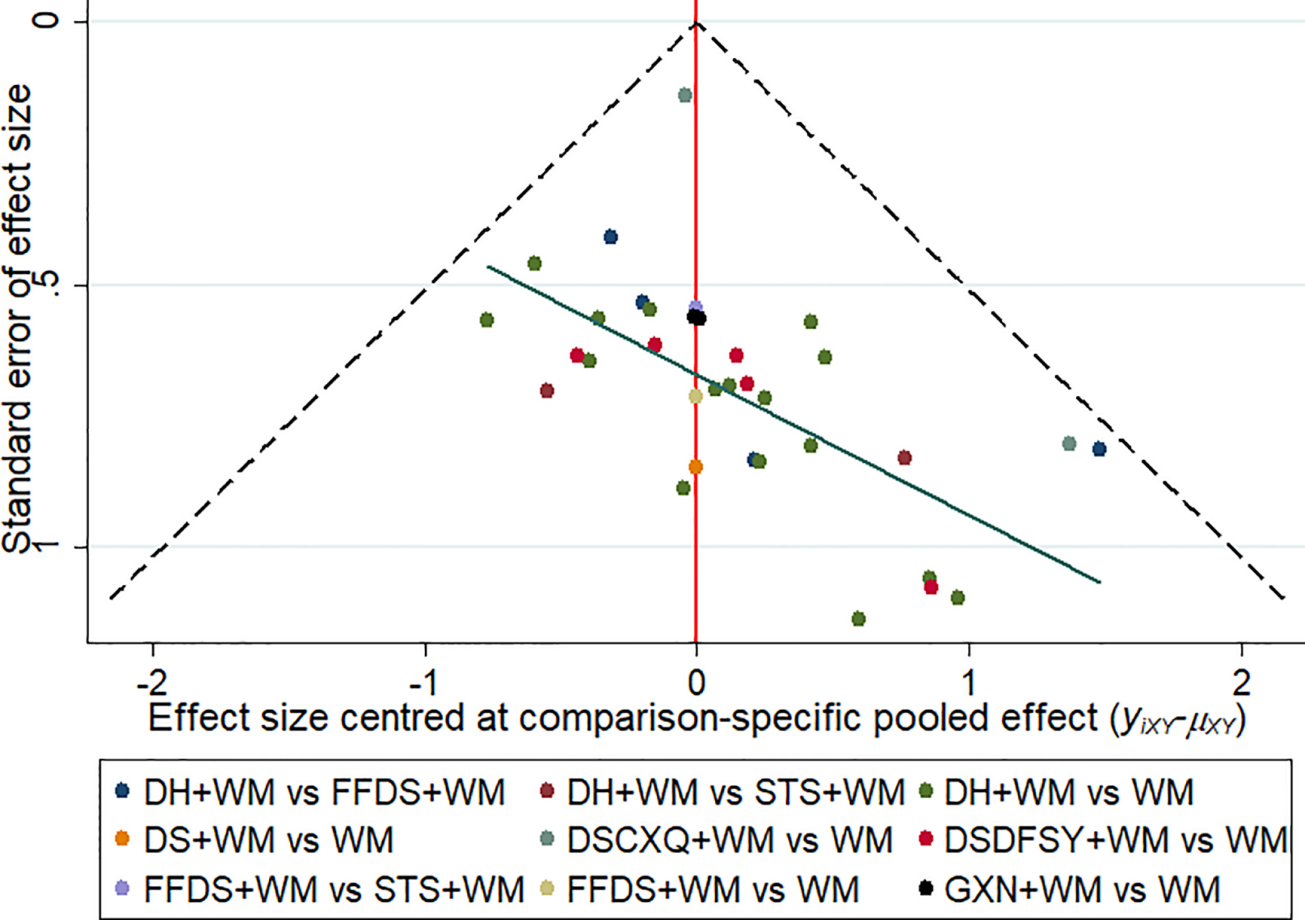
Funnel plots of clinical effectiveness rate. The vertical axis represents “standard error of effect size” and horizontal axis represents “effect size centred at comparison-specific pooled effect (y_ixy_-μ_xy_)”. (DS, Danshen injection; FFDS, Fufang Danshen injection; DH, Danhong injection; DSDFSY, Dansenduofensuanyan injection; DSCXQ, Danshenchuanxiongqin injection; STS, Sodium Tanshinone IIA Sulfonate injection; GXN, Guanxinning injection; WM, western medicine).

This study drew a Comparison—adjusted funnel plot for clinical effectiveness rate to test publication bias. When the distribution points in the funnel chart are symmetrical, it means that there is no publication bias (Riley et al., 2011). As depicted in **Figure 6**, the points in the funnel chart were asymmetric based on the position of the center line, and the angle between the adjusted auxiliary line and the center line was large, indicating a potential publication bias in this study.

### Consistency Test

**Figure 7:**
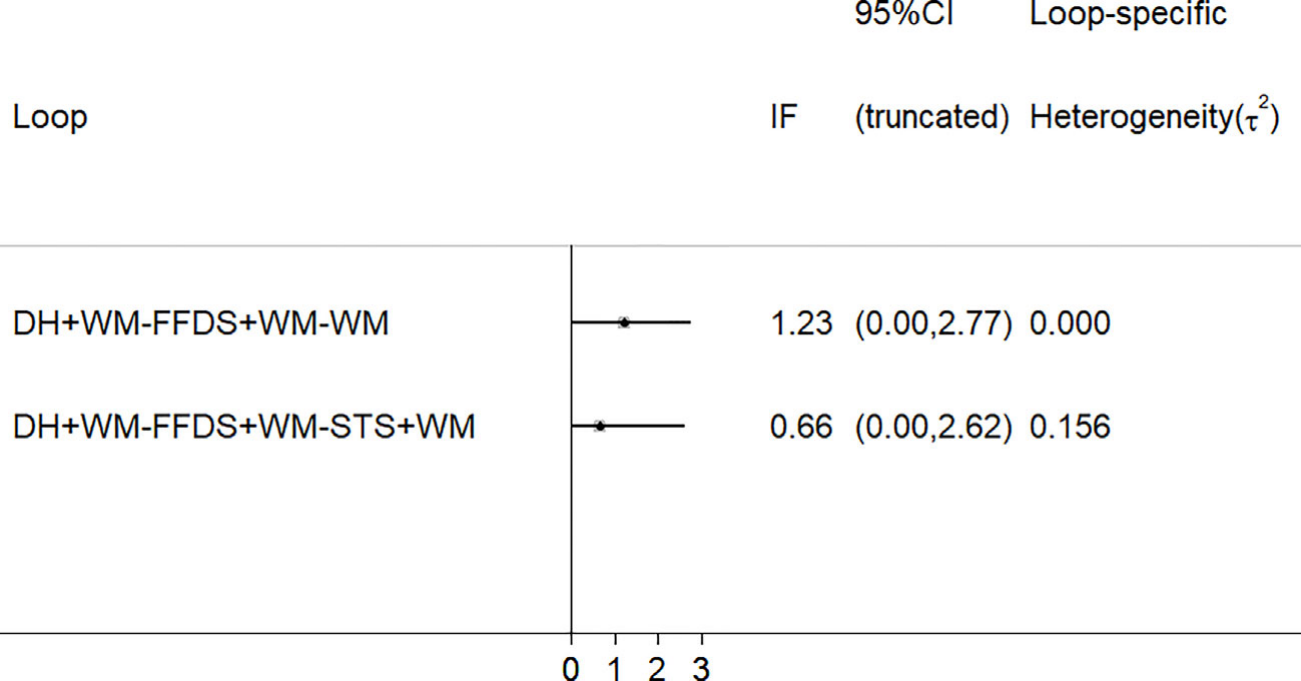
Schematic diagram of consistency. (FFDS, Fufang Danshen injection; DH, Danhong injection; STS, Sodium Tanshinone IIA Sulfonate injection; WM, western medicine).

As depicted in **Figure 7**, there were 2 three-side rings. The results indicated that the 95% CIs contained 0, and IF was between 0.66 and 1.23. Consequently, there was some inconsistency in this NMA study.

## Discussion

We performed the approach of current NMA to evaluate the comparative efficacy and safety of reported DSCIs combination with WM in the treatment of ACS. This study included 53 eligible RCTs with 7 CHIs that evaluated the clinical effectiveness rate, the level of hs-CRP, CRP, IL-6, FIB and ADRs after the application of DSCIs plus WM. According to NMA results, compared with WM alone, the 7 types of CHIs combined with WM therapy were associated with significantly improved the therapeutic effect. In addition, the results of the multidimensional cluster analysis demonstrated that DH+WM and DS+WM had better therapeutic effects. In terms of the ranking of SUCRA probabilities, DH + WM was superior to other CHIs for improving the clinical effectiveness rate. However, since different CHIs have different effects and reactions on patients, the clinical selection of CHIs should better improve the efficacy according to the patient’s condition.

DH is a TCM compound preparation extracted from two famous herbs, Danshen (*Radix Salviae Miltiorrhizae, Salvia miltiorrhiza Bunge*) and Honghua (*Carthami Flos, Carthamus tinctorius L*.) (Feng et al., 2020). Since DH launch in 2002, it has been widely used to prevent and treat ACS (Yao et al., 2011). The main active ingredients of DH are flavonoids and phenolic compounds, for example, danshensu, salvianolic acid B, protocatechuic aldehyde, tanshinone IIA, rosmarinic acid and hydroxysafflor yellow A (Xie et al., 2014; Wang et al., 2020). In addition, previous pharmacological studies have demonstrated that DH have the effects of antioxidant, anti-inflammatory, vasodilation and protect cardiac muscle due to its main pharmacological active components (Wan et al., 2011; Jiang et al., 2015). For example, previous studies have demonstrated that salvianolic acid B could strongly inhibit tumor necrosis factor-α-induced nuclear factor-κB (NF-κB) activation in human aortic endothelial cells (Chen et al., 2001). Furthermore, DH combined with conventional therapy for 2 weeks can significantly reduce the plasma levels of plasma fibrinogen C (FIB-C), glucose protein II b/III a receptor complex and hs-CRP in ACS patients after PCI (Chen et al., 2009; Zhao and Jiang, 2011). In one study, the results indicated that DH + WM not only improved symptoms in patients with UA, but also improved some laboratory indicators, such as CRP, N-terminal brain natriuretic peptide and homocysteine (Sun et al., 2014). Currently, one published NMA showed that DH + WM had advantages in improving the total efficacy and electrocardiography of UA (Liu et al., 2018).

DS is one of most commonly used TCM preparation for treating coronary heart disease, heart-stroke and cerebrovascular diseases. Phenolic acids, including danshensu, salvianolic acid A/B, lithospermic acid B and rosmarinic acid, are the main active contents of DS, extracted from Danshen (Zhang et al., 2005). Besides, the most notable effects of phenolic acid in Danshen are antioxidant, anticoagulant, anti-thrombotic and cell protection (Wang et al., 2007; Orgah et al., 2020). A systematic review revealed that DS as one adjuvant treatment of WM for UA could significantly improve the total clinical effectiveness rate. Furthermore, it also could significantly correct T-wave inversion, reduce the level of FIB and adjust blood lipid level (Wu et al., 2017).

Apart from efficacy, the safety of CHIs in the treatment of ACS is also an important issue worthy of consideration by clinicians. In this study, 28 studies did not address ADRs, and 17 studies indicated that there were no significant ADRs. Thus, we could not draw detailed conclusions about the safety information of CHIs. According to the results, the ADRs mainly included allergic rash, vomiting, drowsiness, flushing, mild gastrointestinal reactions and headache. Previous studies had shown that most of the ADRs linked to DHI therapy were mild and moderately severe, with the primary disposition of discontinuation and without other treatment (Li et al., 2015). In addition, it is noteworthy to monitor the ADRs of patients 30 minutes after CHIs injection. Since few studies in this NMA had focused on ADRs. Therefore, more experiments and clinical evidence are needed to verify the safety of these CHIs.

In terms of the design and contents, this NMA study has some particular merits as follows. First, this is the first NMA to evaluate the efficacy and safety of DSCIs plus WM in the treatment of ACS. Second, it strictly established inclusion and exclusion criteria and a comprehensive literature search. Finally, the rankings of eligible CHIs regarding the different outcome indicators provide evidence and recommendation for the clinical selection of medication.

Nevertheless, several potential limitations of the present NMA should be considered. First, all the enrolled RCTs were only performed in Chinese patients, so it is not clear whether the conclusions of this current study are applicable to ACS patients in other countries. Second, the quality of included studies was not high, mainly because most trials did not report detailed information of allocation concealment and blinding. In addition, due to the diversity of WM and the different doses and courses of CHIs, there may have been clinical heterogeneity. For this reason, we believe that the methodological quality of clinical trials should be valued and improved, in order to promote the appropriate use of CHIs. For instance, RCTs should be registered in advance to ensure transparency, and secondly, the test process should be reported in as much detail as possible in the literature. Despite the above limitations, the results of present NMA demonstrated a complete evaluation of the clinical effect in multiple aspects and provided several clinical suggestions of different CHIs for ACS patients.

## Conclusion

In summary, this NMA results showed that DSCIs combined with WM therapy could have a positive influence on patients with ACS. What’s more, compared with WM alone, DSCIs combined with WM therapy might be associated with significantly improved the therapeutic effect. In addition, DH+WM and DS+WM therapies are potentially the preferred treatments for ACS. However, since most eligible RCTs studies did not focus on the monitoring of ADRs, the safety of these CHIs needs to be further explored. Due to several limitations, more large samples, high-quality clinical and multicenter RCTs studies should be tested and verified in the future.

## Data Availability Statement

The raw data supporting the conclusions of this article will be made available by the authors, without undue reservation.

## Author Contributions

SG and JW conceived and designed the study. SG, MN, SJ, and JZ conducted the systematic review and extracted and analyzed the data. WZ and XL performed interpretation of results. SG drafted the initial manuscript. MW and XZ critically reviewed the manuscript for important intellectual content. All authors contributed to the article and approved the submitted version.

## Funding

This work was supported by the Young Scientists Training Program of Beijing University of Chinese Medicine and the National Nature Science Foundation of China (Grant nos. 81673829).

## Conflict of Interest

The authors declare that the research was conducted in the absence of any commercial or financial relationships that could be construed as a potential conflict of interest.

## Abbreviations

ACS, acute coronary syndrome; ADRs, adverse reactions; CHIs, Chinese herbal injections; CNKI, the China National Knowledge Infrastructure Database; CRP, C-reactive protein; DH, Danhong injection; DS, Danshen injection; DSCIs, Danshen class injections; DSCXQ, Danshenchuanxiongqin injection; DSDFSY, Dansenduofensuanyan injection; DSSA, Danshen Salvianolic Acids injection; FFDS, Fufang Danshen injection; FIB, fibrinogen; FIB-C, fibrinogen C; GXN, Guanxinning injection; hs-CRP, hypersensitive C-reactive protein; IL-6, interleukin-6; MACE, major adverse cardiac events; MDs, mean differences; NF-κB, nuclear factor-κB; NMA, network meta-analysis; NSTEMI, non-ST elevation myocardial infarction; ORs, odds ratios; PCI, percutaneous coronary intervention; PRISMA, the Preferred Reporting Items for Systematic Reviews and Meta-Analyses; RCTs, randomized controlled trials; SinoMed, the Chinese Biomedical Literature Database; STEMI, ST elevation myocardial infarction; STS, Sodium Tanshinone IIA Sulfonate injection; SUCRA, surface under the cumulative ranking; TCM, traditional Chinese medicine; UA, unstable angina; VIP, the Chinese Scientific Journals Full-text Database; WM, western medicine.
