# Supplementary material for: Comparative Efficacy of Danshen Class Injections for Treating Acute Coronary Syndrome: A Multidimensional Bayesian Network Meta-Analysis of Randomized Controlled Trials
Source: Front Pharmacol. 2020 Aug 26;11:1260. doi: 10.3389/fphar.2020.01260 (PMC7485145; doi:10.3389/fphar.2020.01260)
Supplement: Supplementary file 2 [file DataSheet_2.docx]

Supplementary file 2: Search strategy for network meta-analysis.

| **PubMed** |
| --- |
| #1 acute coronary syndrome [MeSH Terms]  #2 ((((Acute Coronary Syndromes [Title/Abstract]) OR Coronary Syndrome, Acute [Title/Abstract]) OR Coronary Syndromes, Acute [Title/Abstract]) OR Syndrome, Acute Coronary [Title/Abstract]) OR Syndromes, Acute Coronary [Title/Abstract]  #3 #1 OR #2  #4 Danshen [Title/Abstract]  #5 Compound danshen [Title/Abstract])  #6 Xiangdan [Title/Abstract]  #7 Fufangdanshen [Title/Abstract]  #8 Danhong [Title/Abstract]  #9 Beitong [Title/Abstract]  #10 Salvianolate [Title/Abstract]  #11 Salvianolic acid [Title/Abstract]  #12 Danshenduofensuan [Title/Abstract]  #13 Danshenchuanxiongqin [Title/Abstract]  #14Sodium tanshinone IIA sulfonate [Title/Abstract]  #15 Guanxinning[Title/Abstract]  #16 #4 OR #5 OR #6 OR #7 OR #8 OR #9 OR #10 #11 OR #12 OR #13 OR #14 OR #15  #17 randomized controlled trial [Publication Type]  #18 controlled clinical trial [Publication Type]  #19 randomized [Title/Abstract]  #20 placebo [Title/Abstract]  #21 randomly [Title/Abstract]  #22 trial [Title/Abstract]  #23 groups [Title/Abstract]  #24 "drug therapy" [Subheading]  #25 #17 OR #18 OR #19 OR #20 OR #21 OR #22 OR #23 OR #24  #26 animals [MeSH Terms]  #27 humans [MeSH Terms]  #28 #26 NOT #27  #29 #25 NOT #28  #30 #3 AND #16 AND #29 |
| **Embase** |
| #1 random*  #2 placebo*  #3 doubl* adj blind*  #4 singl* adj blind*  #5 assign*  #6 allocat*  #7 “double-blind procedure”/exp  #8 ”randomized controlled trial”/exp  #9 ”single-blind procedure”/exp  #10 #1 or #2 or #3 or #4 or #5 or #6 or #7 or #8 or #9  #11 ' acute coronary syndrome '/exp  #12 Acute Coronary Syndromes  #13 Coronary Syndrome, Acute  #14 Coronary Syndromes, Acute  #15 Syndrome, Acute Coronary  #16 Syndromes, Acute Coronary  #17 #11 OR #12 OR #13 OR #14 OR #15 OR #16  #18 Danshen  #19 Compound danshen  #20 Xiangdan  #21 Fufangdanshen  #22 Danhong  #23 Beitong  #24 Salvianolate  #25 Salvianolic acid  #26 Danshenduofensuan  #27 Danshenchuanxiongqin  #28Sodium tanshinone IIA sulfonate  #29 Guanxinning  #30 #18 OR #19 OR #20 OR #21 OR #22 OR #23 OR #24 OR #25 OR #26 OR #27 OR #28 OR #29  #31 #10 AND #17 AND #30 |
| **Cochrane Library** |
| #1 acute coronary syndrome [Title Abstract Keyword]  #2 Acute Coronary Syndromes [Title Abstract Keyword]  #3 Coronary Syndrome, Acute [Title Abstract Keyword]  #4 Coronary Syndromes, Acute [Title Abstract Keyword]  #5 Syndrome, Acute Coronary [Title Abstract Keyword]  #6 Syndromes, Acute Coronary [Title Abstract Keyword]  #7 #1 OR #2 OR #3 OR #4 OR #5 OR #6  #8 Danshen [Title Abstract Keyword]  #9 Compound danshen [Title Abstract Keyword]  #10 Xiangdan [Title Abstract Keyword]  #11 Fufangdanshen [Title Abstract Keyword]  #12 Danhong [Title Abstract Keyword]  #13 Beitong [Title Abstract Keyword]  #14 Salvianolate [Title Abstract Keyword]  #15 Salvianolic acid [Title Abstract Keyword]  #16 Danshenduofensuan [Title Abstract Keyword]  #17 Danshenchuanxiongqin [Title Abstract Keyword]  #18Sodium tanshinone IIA sulfonate [Title Abstract Keyword]  #19 Guanxinning [Title Abstract Keyword]  #20 #8 OR #9 OR #10 OR #11 OR #12 OR #13 OR #14 #15 OR #16 OR #17 OR #18 OR #19  #21 randomized controlled trial [Publication Type]  #22 controlled clinical trial [Publication Type]  #23 randomized [Title Abstract Keyword]  #23 placebo [Title Abstract Keyword]  #24 randomly [Title Abstract Keyword]  #25 trial [Title Abstract Keyword]  #26 groups [Title Abstract Keyword]  #27 #21 OR #22 OR #23 OR #24 OR #25 OR #26  #28 #7 AND #20 AND #27 |

**Reference list:**

Cai G., Huang H. C., Xiao X., and Pang L. L. (2011). Effect of Danhong injection on plasma endothelin and C-reactive protein in patients with acute coronary syndrome. *Chin. J. Integr. Med. Cardio-Cerebrovasc.* 9, 394-395.

Cao M. Y., Lv X. H., He Y., and Liao K. (2010). The effect of Danhong injection in hemorheology in old patients with acute coronary syndrome. *Pract. J. Card. Cereb*. *Pneumal Vasc. Dis.* 18, 541-542.

Chen K. L. (2012). Clinical research on treating acute coronary syndrome by Danhong injection. *Clin. J. Chin. Med.* 4, 82-83.

Chen X. G., and Wang H. P. (2011). Clinical observation of Danhong injection in the treatment of senile acute coronary syndrome. *Chin. J. Clin. Res.* 24, 156-157.

Cui S. D. (2008). Clinical observation of 33 cases of acute coronary syndrome treated by Danhong injection. *Chin. J. Integr. Med. Cardio-Cerebrovasc.* 6, 1091.

Ding Y. (2012). Effect of Danhong injection on plasma brain natriuretic peptide in patients with acute coronary syndrome. *J. Emerg. Tradit. Chin. Med.* 21, 281-282.

Du P. (2011). Intervention of Danhong injection on serum MMP-9 in patients with acute coronary syndrome. *China Health Ind.* 8, 68. doi:10.16659/j.cnki.1672-5654.2011.29.052

Du Z. A., and Chen J. L. (2009). Clinical observation of Danhong injection in the treatment of acute coronary syndrome. *J. Med. Forum*. 30, 101-102.

Fei S. B., Guan Y. Y., Zhang L., Lin G., Zhao G., Huang J. F., et al. (2010). Effect of Danhong injection and atorvastatin on the levels of circulating IL -6, sCD40L and APN in acute coronary syndrome. *Chin. J. Integr. Med. Cardio-Cerebrovasc. Dis.* 8, 1035-1037.

Feng Z. H. (2011). The clinical study of Danhong injection on the levels of serum hs-CRP and MMP-9 in patients with acute coronary syndromes. *China Health Ind.* 8, 16-17. doi: 10.16659/j.cnki.1672-5654.2011.29.048

Gu Y. F., Zhang X., Jin J., Xue S. F., Chen J. F., Fan C. F., et al. (2011). The effect of Danhong injection on serum high sensitivity C-reaction protein in patients with acute coronary syndrome. *Chin. J. Cardiovasc. Res.* 9, 357-359.

Guan Y. Y., Fei S. B., Wang Y. Sheng Z. Q., Shi L. S., Zhang L., et al. (2010). Effect of Danhong injection on circulating inflammatory factors and TXB2 in acute coronary syndrome. *Chin. J. Integr. Med. Cardio-Cerebrovasc. Dis.* 8, 1039-1040.

He Y. L. (2014). Clinical analysis of Danhong injection applied to treat acute coronary artery syndrome. *J. Sichuan Tradit. Chin. Med.* 32, 165-167.

Hong Y. D., Wu H., Mo H. H., and Li F. Y. (2004). Effects of Xiangdan injection in acute coronary syndrome and inflammatory markers. *Tradit. Chin. Drug Res. Clin. Pharmacol.* 15, 425-428. doi: 10.19378/j.issn.1003-9783.2004.06.019

Hou X. J. (2013). Comparative observation of the efficacy of Danhong injection and Tanshinone IIa sulfonate injection in the treatment of 99 cases of acute coronary syndrome. *Asia-Pacific Tradit. Med*. 9, 163-164.

Lao G. H., Chen G. Y., Li H. D., and Chen J. (2013). Lipid-lowering effect of Danhong injection combined with low-dose simvastatin on acute coronary syndrome. *Pract. Clin. Med.* 14, 10-11.

Li A. X., Jin M. H., Li D., and Zhang Li. (2019). Clinical effect of Danshen polyphenolate injection combined with ticagrelor in the treatment of patients with acute coronary syndrome. *Heilongjiang Med. Pharm.* 42, 51-52.

Li C. L., Huang H. G., and Deng P. (2007). Effect of Danhong injection on plasma tissue factors in patients with acute coronary syndrome. *J. Chin. Physician.* 9, 984-985.

Li J. H. (2013). Salvia miltiorrhiza polyphenolate injection combined with low molecular weight heparin calcium in the treatment of 46 cases of acute coronary syndrome. *Nei Mongol J. Tradit. Chin. Med*. 32, 2-3. doi: 10.16040/j.cnki.cn15-1101.2013.26.158

Li M., Chen X. C., Song W. D., Wang X. Y., Wei D. Y., Qin H. Q., et al. (2016). Effect of Guanxining Injection on serum YKL-40 and hs-CRP levels in patients with non-ST-segment elevation acute coronary syndrome. *J. North Pharm.* 13, 142-143.

Li S. P. (2015). Clinical observation of Danhong injection in the treatment of senile acute coronary syndrome. *Med. Forum*. 19, 1328-1329.

Li W. H., Zhang Y. B., Zhu H., Xu T. D., Wang Z. R., Qian W. H., et al. (2010). Effect of Danhong injection on the high-sensitivity C-reactive protein, fibrinogen and von willebrand factor in patients with acute coronary syndrome. *Chin. J. Gen. Pract.* 8, 1222-1223. doi: 10.16766/j.cnki.issn.1674-4152.2010.10.010

Li Y. H., and Ge H. Y. (2011). Observation on treatment of acute coronary syndrome by Danhong injection. *J. Med. Forum.* 32, 150-152.

Li Y. L., and Meng Z. H. (2008). Danhong injection was used to treat 48 cases of acute coronary syndrome. *J. Pract. Tradit. Chin. Intern. Med*. 22, 20-21. doi: 10.13729/j.issn.1671-7813.2008.03.010

Li Y. M., Chen X. H., Li J., Hu J. P., Bian Y. L., and Wu Y. J. (2007). Clinical study of Danshen injection in treating acute coronary syndrome. *Chin. J. Tradit. Med. Sci. Tech*. 14, 392-393.

Li Y. N., and Song X. (2011). Effect of Danhong injection on serum hypersensitive C-reactive protein in patients with acute coronary syndrome. *Chin. J. Mod Drug Appl.* 5, 167-168. doi: 10.14164/j.cnki.cn11-5581/r.2011.03.018

Liang J., Zhang Z. L., Wang Z. J., and Zhang K. C. (2019). Effects of Danhong injection on inflammatory factors vascular endothelial function and platelet activation in patients with acute coronary syndrome. *Chin. J. Integr. Med. Cardio-Cerebrovasc. Dis.* 17, 1999-2001.

Liao Y. J., Liu C. H., Ping Z. X., Jiang T., and Yao J. (2013). Effect of Guanxining injection on plasma fibrinogen and C-reactive protein in patients with acute coronary syndrome. *Guide China Med.* 11, 698-699.

Lin S. L. (2015). Effect of Salvia Miltiorrhiza Polyphenolate on the level of modified albumin in angina pectoris. *Chin. J. Urban Rural Enterp. Hyg*. 30, 110-111. doi: 10.16286/j.1003-5052.2015.02.047

Lin Y. C., and Yu Z. S. (2017). Influence and its efficacy of salvianolate adjuvant therapy on vascular endothelial function in patients with acute coronary syndrome. *China Mod. Doct.* 55, 26-28.

Liu X., Wang Y., and Jin J. (2008). Clinical analysis of 300 cases of acute coronary syndrome treated by Danhong injection. *Pract. J. Card. Cereb*. *Pneumal Vasc. Dis.* 16, 24-25.

Long T. X. (2011). Study on the clinical efficacy of Danshen injection in the treatment of acute coronary syndrome. *Chin. J. Clin. Ration. Drug Use*. 4, 44-45. doi: 10.15887/j.cnki.13-1389/r.2011.27.006

Ma J., and Liang X. Y. (2016). Effect of Danshen injection on serum homocysteine, folic acid and C-reactive protein levels in patients with ST-segment elevation acute myocardial infarction. *Chin. J. Integr. Med. Cardio-Cerebrovasc. Dis.* 14, 2528-2530.

Nie L. S., and Zhao N. J. (2012). Clinical observations of 60 patients regarding the effect of danhong injection on hs-CRP of patients with acute coronary syndrome. *World Health Dig.* 9, 409-410. doi: 10.3969/j.issn.1672-5085.2012.16.397

Ruan L., Jiao X. M., Li J., Wang C. L., and Li T. (2017). Effects of Xiangdan injection on serum levels of C-reactive protein, amino terminal brain natriuretic peptide and cardiac enzymes of patients with acute coronary syndrome. *Prog. Mod. Biomed.* 17, 3481-3485. doi: 10.13241/j.cnki.pmb.2017.18.018

Shi K. (2013). Treating cute coronary syndrome with Danshen. *Clin. J. Chin. Med*. 5, 23-24.

Su H. C., and Zhao C. (2017). Effect of Danshen injection on serum homocysteine and C reactive protein in patients with ST segment elevation acute myocardial infarction. *J. Snake*. 29, 415-416.

Su K., and Jiang Y. B. (2012). Effect of Danhong injection on serum high sensitivity C-reactive protein in patients with acute coronary syndrome. *China Pract. Med*. 7, 22-23. doi: 10.14163/j.cnki.11-5547/r.2012.25.095

Wang J. X., Xie Y. Q., Guo C. F., Gao H. Y., Peng H., Chen Y. Q., et al. (2015). Clinical observation of 245 cases of elderly patients with acute coronary syndrome treated by Danhong injection. *World Chin. Med.* 10, 1.

Wang S. P., and Zhang A. P. (2012). Clinical observation of Danhong injection in treating senile acute coronary syndrome. *J. Emerg. Tradit. Chin. Med*. 21, 1181-1182.

Wei Y. S. (2012). Effect of Danhong injection on plasma endothelin-1 and high sensitivity C-reactive protein in patients with acute coronary syndrome. *China Mod. Med.* 19, 31-32.

Wu G. P., Huang X. H., and Yao Z. (2009). Influence of danshen ligustrazin for injection on serum high-sensitivity C-reactive protein and cardiac function of patients with acute coronary syndrome. *J. Hainan Med. Uni.* 15, 858-860. doi: 10.13210/j.cnki.jhmu.2009.08.004

Xia L. J., Chen W. D., and Wu Y. M. (2017). Clinical observation on curative effect of acute coronary syndrome treated with salvianolate injection and its influences on serum hs-CRP, IL-6 levels. *Chin. J. Tradit. Med. Sci. Tech*. 24, 4-5.

Xia S. L., and Wang Y. (2011). Effects of Guanxinning injection on non-ST-segment elevation acute coronary syndrome. *China Mod. Doct.* 49, 99-100.

Xiao M. S. (2009). Comparision of the curative effects of danhong injection VS compound danshen injection in the treatment for the elderly patients with non-ST-elevation acute coronary syndrome. *J. Pract. Electrocardiol.* 18, 19-21. doi: 10.13308/j.issn.1008-0740.2009.01.003

Xu L. C. (2011). Clinical observation of 42 cases of acute coronary syndrome treated by Danhong injection combined with western medicine. *Nei Mongol J. Tradit. Chin. Med.* 30, 71-72. doi: 10.16040/j.cnki.cn15-1101.2011.21.123

Yang L. J. (2010). Effect of Danhong injection on plasma endothelin in patients with acute coronary syndrome. *Mod. J. Integr. Tradit. Chin. West. Med*. 19, 51-52.

Yang M. J., and Zhang B. S. (2010). Cost-effectiveness analysis of two regimens for acute coronary syndrome. *Ningxia Med. J.* 32, 1252-1253. doi: 10.13621/j.1001-5949.2010.12.037

Zhang S. G., He L. M., Liu S. Y., Li Q. Y., and Tan R. R. (2012). Clinical observation on efficacy and safety of traditional Chinese medicine for activating blood circulation and removing blood stasis in acute coronary syndrome. *China & Foreign Med. Treat.*31, 4-6. doi: 10.16662/j.cnki.1674-0742.2012.13.011

Zhang S., Shi P., and Ma L. (2014). Evaluation of Danshen ligustrazine on improving the clinical symptoms of non-ST elevation acute coronary syndrome (middle and low risk group). *China Pract. Med*. 9, 143-145. doi: 10.14163/j.cnki.11-5547/r.2014.25.098

Zhang Z. C., Chang X. G., Xu Y., and Zhang L. Q. (2010). Effects of DANHONG injection on hs-CRP, IL6, TC, TG and LDL-C in old patients with acute coronary syndrome. *Chin. Arch. Tradit. Chin. Med.* 28, 2237-2238. doi: 10.13193/j.archtcm.2010.10.223.zhangzhch.033

Zhao Q. F. (2011). Observation on the curative effect of Guanxining injection in treating acute coronary syndrome. *Chin. J. Misdiagnostics*. 11, 7864-7865.

Zhou K. J. (2011). The effect of Danhong injection on the level of serum hs-CRP in patients with acute coronary syndromes. *China Health Ind.* 8, 11-12. doi: 10.16659/j.cnki.1672-5654.2011.29.046
