# Supplementary material for: Comparative Efficacy of Danshen Class Injections for Treating Acute Coronary Syndrome: A Multidimensional Bayesian Network Meta-Analysis of Randomized Controlled Trials
Source: Front Pharmacol. 2020 Aug 26;11:1260. doi: 10.3389/fphar.2020.01260 (PMC7485145; doi:10.3389/fphar.2020.01260)
Supplement: Supplementary file 3 [file DataSheet_3.docx]

**Supplementary file 3:** **Detailed information on included CHIs.**

| **Chinese herbal injection** | **Source** | **Species / Raw materials** | **Botanical plant names** | **Phytochemical compositions** | **Chemical composition criteria** | **Therapeutic claims in TCM** | **Indications** | **Quality control reported? (Y/N)** | **Chemical analysis reported? (Y/N)** |
| --- | --- | --- | --- | --- | --- | --- | --- | --- | --- |
| Danshen injection | Sichuan Shenghe Pharmaceutical Co., Ltd./ Shanghai Zhongxi Pharmaceutical Co., Ltd./ Zhengda Qingchunbao Medicine Industry | *SALVIAE MILTIORRHIZAE RADIX ET RHIZOMA* (Dan Shen) 1500g. | Root or rhizome of *Salvia miltiorrhiza Bunge.* | Salvianolic acid A, salvianolic acid B, Dan shensu, protocatechuic aldehyde, rosmarinic acid, lithospermic acid B and et al. | Danshensu  (C_9_H_10_O_5_) > 0.60 mg/mL; salvianolic acid B (C_36_H_30_O_16_) > 0.10 mg/mL; protocatechuic aldehyde (C_7_H_6_O_3_) >0.2mg/mL. | Activating blood circulation and removing blood stasis. | Coronary heart disease, cardiovascular disease and angina pectoris and et al. | Y - National Food and Drug Administration National Drug Standards.  Standard number: WS3-B-3766-98. | Y- HPLC-UV and HPLC-MS |
| Fufang Danshen injection | Zhengda Qingchunbao Medicine Industry | *SALVIAE MILTIORRHIZAE RADIX ET RHIZOMA* (Dan Shen) 100g and *DALBERGIAE ODORIFERAE LIGNUM* (Jiang Xiang) 1000g | Root or rhizome of *Salvia miltiorrhiza Bunge and* dried heartwood of *Dalbergia odorifera T. Chen.* | Salvianolic acid B, protocatechuic aldehyde, propanoid acid, rosmarinci acid, and et al. | Protocatechuic aldehyde (C_7_H_6_O_3_) > 0.17mg/mL. | Promoting blood flow to dissipate stasis and regulating the wide chest. | Coronary diseases, heart diseases, cerebral vascular diseases and et al. | Y - National Food and Drug Administration National Drug Standards.  Standard number: WS3-B-3289-98 | N |
| Danhong injection | Buchang Pharmaceutical Co., Ltd. | *SALVIAE MILTIORRHIZAE RADIX ET RHIZOMA* （Dan Shen）750g and *CARTHAMI FLOS* (Hong Hua) 250g. | Root or rhizome of *Salvia miltiorrhiza Bunge and* dried flower of *Carthamus tinctorius L.* | Danshensu, salvianolic acid B, protocatechuic aldehyde, tanshinone IIA, rosmarinic acid hydroxysafflor yellow A and et al. | Each 1ml of this product contains salvia miltiorrhizae based on the total amount of danshensu (C_9_H_10_O_5_) and protocatechuic aldehyde (C_7_H_6_O_3_), not less than 0.5mg. Each 1ml contains total flavonoids as rutin (C_27_H_30_O_16_), not less than 5.0mg. | promoting blood flow to dissipate stasis and clearing meridians. | Coronary heart disease, angina pectoris, myocardial infarction, ischemic encephalopathy and et al. | Y - National Food and Drug Administration National Drug Standards.  Standard number: WS-11221(ZD-1221)-2002 | Y- UHPLC/  orbitrap-MS |
| Dansenduofensuanyan injection | Shanghai Green Valley Pharmaceutical Co., Ltd. | *SALVIAE MILTIORRHIZAE RADIX ET RHIZOMA* （Dan Shen）and salvianolate. | Root or rhizome of *Salvia miltiorrhiza Bunge.* | Magnesium lithospermate B, etc. | This product contains more than 80% of magnesium lithospermate B (C_36_H_28_MgO_16_). | promoting blood flow to dissipate stasis and clearing meridians. | Angina, myocardial infarction, ischemic stroke and et al. | Y - National Food and Drug Administration National Drug Standards.  Standard number: YBZ09012005-2010Z | N |
| Danshenchuanxiongqin injection | Guizhou Beit Pharmaceutical Co., Ltd. | *SALVIAE MILTIORRHIZAE RADIX ET RHIZOMA* （Dan Shen） 200g，ligustrazine hydrochloride 20g and glycerol 200mL. | Root or rhizome of *Salvia miltiorrhiza Bunge.* | Ligustrazine hydrochloride, danshensu and et al. | Each 1ml of this product contains Danshen as Danshensu (C_9_H_10_O_5_) should be 0.36 ~0.44mg. | Activating blood circulation and removing blood stasis. | Coronary heart disease angina, myocardial infarction, ischemic stroke, etc. | Y - National Food and Drug Administration National Drug Standards.  Standard number: WS-10001-(HD-1138)-2002 | N |
| Sodium Tanshinone IIA Sulfonate injection | Shanghai First Biochemical Pharmaceutical Co., Ltd. | *SALVIAE MILTIORRHIZAE RADIX ET RHIZOMA* （Dan Shen） | Root or rhizome of *Salvia miltiorrhiza Bunge.* | Sodium tanshinone IIA sulfonate, etc. | The sodium tanshinone IIA sulfonate (C_19_H_17_NaO_6_S) should be 90.0% ~ 110.0% of the marked amount. | Activating blood circulation and removing blood stasis. | Coronary heart disease angina, myocardial infarction, hypertensive nephropathy, etc. | Y - National Food and Drug Administration National Drug Standards.  Standard number: WS-10001-(HD-1014)-2002 | Y- HPLC-DAD and liquid chromatography/multistage |
| Guanxinning injection | Yabao Pharmaceutical Group Co., Ltd. | *SALVIAE MILTIORRHIZAE RADIX ET RHIZOMA* （Dan Shen）2000g and *CHUANXIONG RHIZOMA* (Chuan Xiong) 2000g. | Root or rhizome of *Salvia miltiorrhiza Bunge and* root of *Ligusticum chuanxiong Hort.* | protocatechuic aldehyde, etc. | Protocatechuic aldehyde (C_7_H_6_O_3_) > 0.30mg. | Activating blood circulation and removing blood stasis. | Coronary heart disease angina, and so on. | Y - National Food and Drug Administration National Drug Standards.  Standard number: WS3-B-3267-98 | N |

**References**

Shao H. K., Li M. S., Chen F. C., Chen L. H., Jiang Z. J., Zhao L. G. (2018). The Efficacy of Danshen Injection as Adjunctive Therapy in Treating Angina Pectoris: A Systematic Review and Meta-Analysis. *Heart Lung Circ*. 27, 433-442. doi: 10.1016/j.hlc.2017.10.016.

Zhang J. L., Cui M., He Y., Yu H. L., Guo D. A. (2005). Chemical fingerprint and metabolic fingerprint analysis of Danshen injection by HPLC-UV and Q14 HPLC-MS methods. *J. Pharm. Biomed Anal*. 36, 1029-35. doi: 10.1016/j.jpba.2004.09.009.

Yuan T. H., Chen Y., Zhou X. Q., Lin X. Y., Zhang Q. S. (2019). Effectiveness and Safety of Danshen Injection on Heart Failure: Protocol for a Systematic Review and Meta-Analysis. *Medicine (Baltimore)* 98, e15636. doi: 10.1097/MD.0000000000015636.

Shen Z. B., Yin Y. Q., Tang C. P., Yan C. Y., Chen C., Guo L. B. (2010). Pharmacodynamic Screening and Simulation Study of Anti-Hypoxia Active Fraction of Xiangdan Injection. *J. Ethnopharmacol.* 127, 103-7. doi: 10.1016/j.jep.2009.09.041.

Feng C., Wan H., Zhang Y., Yu L., Shao C., He Y., et al. (2020). Neuroprotective Effect of Danhong Injection on Cerebral Ischemia-Reperfusion Injury in Rats by Activation of the PI3K-Akt Pathway. *Front. Pharmacol*. 11, 298. doi: 10.3389/fphar.2020.00298.

Xu L. L., Shang Z. P., Bo T., Sun L., Guo Q. L., Qiao X., et al. (2019). Rapid Quantitation and Identification of the Chemical Constituents in Danhong Injection by Liquid Chromatography Coupled with Orbitrap Mass Spectrometry. *J. Chromatogr. A*. 1606, 460378. doi: 10.1016/j.chroma.2019.460378

Liu S., Wu J., Zhang D., Tan D. (2018). What are the best Salvia miltiorrhiza injection classes for treatment of unstable angina pectoris? A systematic review and network Meta-analysis. *J. Tradit. Chin. Med*. 38, 321-338.

Zhang X. M., Wu J. R., Zhang B., Zhou W. (2016). Danshenchuanxiongqin Injection in the Treatment of Unstable Angina Pectoris: A Systematic Review and Meta-analysis. *J. Tradit. Chin. Med.* 36, 144-50. doi: 10.1016/s0254-6272(16)30020-6.

Chen S. Q., Zhao X., Li Y., Yang D., Zhou T. T., Fan G. (2013). Impurities Preparation of Sodium Tanshinone IIA Sulfonate by High-Speed Counter-Current Chromatography and Identification by Liquid Chromatography/Multistage Tandem Mass Spectrometry. *J. Chromatogr. A.* 1288, 28-34. doi: 10.1016/j.chroma.2013.02.055

Xu J. Y., Zhang C. H., Shi X. Q., Li J., Liu M., Jiang W. M., et al. (2019). Efficacy and Safety of Sodium Tanshinone IIA Sulfonate Injection on Hypertensive Nephropathy: A Systematic Review and Meta-Analysis. *Front. Pharmacol*. 10, 1542. doi: 10.3389/fphar.2019.01542. eCollection 2019.

Jia Y. L., Leung S. W., Lee M, Y, Cui G. Z., Huang X. H., Pan F. H. (2013). The Efficacy of Guanxinning Injection in Treating Angina Pectoris: Systematic Review and Meta-Analysis of Randomized Controlled Trials. *Evid. Based Complement Alternat. Med*. 2013, 282707. doi: 10.1155/2013/282707.
